# A cost-effectiveness analysis of provider and community interventions to improve the treatment of uncomplicated malaria in Nigeria: study protocol for a randomized controlled trial

**DOI:** 10.1186/1745-6215-13-81

**Published:** 2012-06-09

**Authors:** Virginia Wiseman, Ezeoke Ogochukwu, Nwala Emmanuel, Mangham Lindsay J, Cundill Bonnie, Enemuo Jane, Uchegbu Eloka, Uzochukwu Benjamin, Onwujekwe Obinna

**Affiliations:** 1 Department of Global Health and Development, London School of Hygiene and Tropical Medicine, London, UK; 2 Health Policy Research Group, Department of Pharmacology and Therapeutics, College of Medicine, University of Nigeria (Enugu Campus), Enugu, Nigeria; 3 Department of Infectious Disease Epidemiology, London School of Hygiene and Tropical Medicine|, London, UK

**Keywords:** Cost-effectiveness, Malaria, Rapid Diagnostic Tests, Interventions, Guidelines, Economics

## Abstract

**Background:**

There is mounting evidence of poor adherence by health service personnel to clinical guidelines for malaria following a symptomatic diagnosis. In response to this, the World Health Organization (WHO) recommends that in all settings clinical suspicion of malaria should be confirmed by parasitological diagnosis using microscopy or Rapid Diagnostic Test (RDT). The Government of Nigeria plans to introduce RDTs in public health facilities over the coming year. In this context, we will evaluate the effectiveness and cost-effectiveness of two interventions designed to support the roll-out of RDTs and improve the rational use of ACTs. It is feared that without supporting interventions, non-adherence will remain a serious impediment to implementing malaria treatment guidelines.

**Methods/design:**

A three-arm stratified cluster randomized trial is used to compare the effectiveness and cost-effectiveness of: (1) provider malaria training intervention versus expected standard practice in malaria diagnosis and treatment; (2) provider malaria training intervention plus school-based intervention versus expected standard practice; and (3) the combined provider plus school-based intervention versus provider intervention alone. RDTs will be introduced in all arms of the trial. The primary outcome is the proportion of patients attending facilities that report a fever or suspected malaria and receive treatment according to malaria guidelines. This will be measured by surveying patients (or caregivers) as they exit primary health centers, pharmacies, and patent medicine dealers. Cost-effectiveness will be presented in terms of the primary outcome and a range of secondary outcomes, including changes in provider and community knowledge. Costs will be estimated from both a societal and provider perspective using standard economic evaluation methodologies.

**Trial registration:**

Clinicaltrials.gov NCT01350752

## Background

### Rationale for the study

In 2001, as a response to increasing levels of resistance to antimalarial medicines, the World Health Organization (WHO) recommended that all countries experiencing resistance to conventional monotherapies should use combination therapies, preferably those containing artemisinin derivatives (artemisinin-based combination therapies (ACTs)) for the treatment of uncomplicated *P. falciparum* malaria [1,2].

The switch to ACTs raises a number of challenges not least of which is their relatively high cost. For many countries, ACTs are as much as 10 times the price of most monotherapies [3-5]. Combined with the fact that clinical diagnosis may result in over-diagnosis because the signs and symptoms of malaria are non-specific and therefore overlap with other febrile diseases [6], then ‘the relatively high cost of ACTs makes waste through unnecessary treatment of patients without parasitaemia unsustainable’ [7].

This has led to growing pressure to improve the specificity of malaria diagnosis. In 2010, the WHO released a second edition of *Guidelines for the Treatment of Malaria* in which it recommends parasitological confirmation of suspected malaria cases in all patients before treatment where testing facilities are available [8]. In addition to securing cost savings, it is argued that parasitological diagnosis: improves patient care in parasite-positive patients owing to greater certainty that the patient has malaria; helps to identify parasite-negative patients in whom another diagnosis must be sought; prevents unnecessary exposure to antimalarials, thereby reducing side-effects, drug interactions, and selection pressure; improves health information; and confirms treatment failures [7].

Two methods for parasitological testing of malaria are microscopy and Rapid Diagnostic Tests (RDTs). While microscopy has been the gold standard, a number of strong arguments have been put forward in favor of RDTs. RDTs have the potential to provide accurate and timely diagnosis to those previously unable to access good quality microscopy services [4,8-11]. They are relatively simple to use and do not require specialized skills [12,13] compared to microscopy, which is labor-intensive and time-consuming [9]. They do not require laboratory equipment and reagents that are often unavailable in remote locations or resource-poor settings [11].

According to the WHO, the move towards universal diagnostic testing of malaria is a critical step forward in the fight against malaria as it will allow for the targeted use of ACTs for those who actually have malaria. In practice however, studies suggest that there are persistent barriers to universal testing. A distrust of test results particularly negative ones [14-16], lack of alternative drugs with which to treat fever patients [17,18], and patient demand for inappropriate medicines [17,19-21] are some of the factors shown to influence whether a malaria test is done and in turn, acted upon.

Our own formative research in Nigeria has also revealed barriers to the effective implementation of malaria treatment guidelines [19]. Between May 2009 and June 2010 using cross-sectional cluster surveys with patients and providers, and a series of focus group discussions, it was shown that very few facilities had malaria testing available; no medicine retailers and only 13% of public facilities had microscopy available and none had RDTs. Despite ACTs becoming the recommended treatment for uncomplicated malaria in Nigeria in 2005, they remain underused. Approximately 80% of health facilities (including medicine retailers) had ACTs in stock at the time of the survey but only 55% of providers (by which we mean health workers) knew that ACTs are the recommended treatment. ACTs were received by only 22% of fever cases treated at health facilities, and were often received in the wrong dose (34%). Sulfadoxine-pyrimenthamine (SP) is no longer recommended for treating malaria but was still frequently used with just over one-third of patients receiving this medicine. Our research also highlighted the importance of patient demand in influencing the treatment received. Approximately 55% of patients surveyed requested a specific medicine and in most cases this was not the nationally recommended treatment. Providers appear to be influenced by what they perceive patients to want or are able to afford. There was also considerable skepticism among patients and caregivers about negative test results, highlighting the need for greater awareness of alternative causes of febrile illness.

These findings reinforce the need to ensure that the large-scale roll-out of RDTs in Nigeria is accompanied by interventions that encourage providers to deliver treatment consistent with guidelines. While there have been several evaluations assessing whether the introduction of RDTs will be cost-effective compared to both presumptive treatment and to field microscopy [4,22-24], surprisingly little evidence exists about the cost-effectiveness of training interventions to support the large scale roll-out of RDTs [25]. This study will use a cluster randomized design, in public facilities and medicine retailers, to compare the cost-effectiveness of a provider training intervention and a combined provider training and school-based malaria intervention to expected standard practice of supplying RDTs with a demonstration on how to use them. The overall aim is to assist Nigerian policymakers in their pursuit of delivering maximum health benefits and value for money in malaria control.

## Methods/design

The interventions will be evaluated using a three-arm stratified, cluster randomized trial across 42 clusters, 14 clusters per arm, in two areas in Nigeria. Since the school-based intervention is being delivered at the community level a cluster is defined as a geographical community which contains at least one facility and one school, and this will be the unit of randomization with study site (urban-rural setting) as the stratum. Clusters will be selected at random within each stratum with the number per stratum selected probability proportional to size. Due to logistics and costs, a total of 138 health facilities and 38 schools will be included in the study. Schools and facilities will be randomly selected from within each cluster to receive the cluster intervention. Up to three schools per cluster will be randomly selected from a list of schools provided by the Research and Statistics Department of the Ministry of Education Enugu State, while the number of facilities per cluster will be selected probability proportional to size. Communities will be randomized to one of the following three arms:

Arm 1 (expected standard practice):

Facilities invited to supply RDTs

Demonstration on how to use RDTs

Arm 2

Facilities invited to supply RDTs

Provider intervention: training and supervision on malaria diagnosis and treatment (which includes a demonstration on how to use RDTs)

Arm 3

Facilities invited to supply RDTs

Provider intervention: training and supervision on malaria diagnosis and treatment (which includes a demonstration on how to use RDTs)

School-based malaria education intervention.

The first arm represents expected standard practice when RDTs are introduced in public health facilities and medicine retailers. This is the approach most likely to be adopted by the State Malaria Control Programme and the Association of Community Pharmacists and Association of PMDs in the near future. All of these organizations have been involved in the design of the interventions. To date, only a small quantity of RDTs has been distributed with basic training to public health facilities in Nigeria. None have been distributed to facilities taking part in this study.

Outcomes will be assessed through exit interviews with patients as well as provider surveys and household surveys. Economic and financial costs will also be measured to enable the calculation of incremental cost-effectiveness ratios. Ethical approval for this study has been obtained from the University of Nigeria and the London School of Hygiene and Tropical Medicine.

### Study area

The study is being conducted in two sites in Enugu State in south-eastern Nigeria. Enugu state is geographically located in the southern zone of Nigeria between 7°10′N and 7°45′N of the Equator and on longitude of 7.4878°E and latitude of 6.4231°N. It is bordered on the north by Kogi and Benue States and on the south by Abia and Imo States, on the east by Ebonyi State, and on the west by Anambra State. The bioclimatic zone is rainforest in nature with annual rainfall between 152 cm and 203 cm and temperature ranges from 22.2°C to 30.6°C. The state has a land area of 7,617.82 km^2^ and a population of 3,289,589 people. The activities of the majority of the population include farming, fishing, wine tapping, and poultry keeping and rearing of domestic animals. The main occupation which is farming runs from November to February. The people of Enugu are of Igbo ethnicity and speak the Igbo language.

The two sites are Enugu Urban (comprising of Enugu East, Enugu South, and Enugu North) and Udi Local Government Areas (LGA). Enugu Urban is the largest predominantly urban area in Enugu State and contains a population of 722,664 (National Bureau of Statistics: http://www.nigerianstat.gov.ng). However, about 30% of Enugu East LGA is rural. The Udi study site lies to the west of Enugu and is predominantly rural. The population of the Udi LGA is estimated to be 234,002 (National Bureau of Statistics: http://www.nigerianstat.gov.ng). The land mass of Udi LGA is more than that of the combined three component LGAs of Enugu Urban.

Malaria is endemic in Enugu state, and occurs all year round. Research in the study area shows that patent medicine dealers (PMDs, also known as patent medicine vendors) are the major source of treatment for malaria [26-29]. These studies also show that chloroquine, SP, and artesunate monotherapy are still provided and consumed for the treatment of malaria.

The study sites are similar in terms of language and culture but differ in terms of number of health facilities, due to the rural nature of Udi LGA, which has fewer public facilities and pharmacies while the reverse is the case in Enugu which is predominantly urban.

The communities are autonomous and all have a traditional ruler. Hence, a distinct community will have a traditional ruler and in some cases, a town union executive council. Most communities are comprised of component villages and the numbers of villages in a community depends on the size of the community. Each village is in turn comprised of super-family units that trace their origin to a common progenitor. The super-family units are made up of a number of households. All communities have at least one primary school and most have a secondary school.

### Participants

Interviewers will explain to all participants that involvement in the study is voluntary and they have the right to withdraw at any point in time and to ask any questions. Information about the study will be read to all participants and provided in hard copy. All consenting participants will be asked to sign two standard consent forms (that is one for the patient to take home and one retained by the interviewer).

#### Health facilities

Two types of healthcare facilities are included in the study: public primary health facilities and medicine retailers (including private pharmacies and private PMDs).

Public primary health facilities include primary health centers, dispensaries, and health posts. They are expected to provide healthcare services for the prevention and treatment of common endemic diseases. There are rarely laboratory services at this level. Nurses, senior and junior community health extension workers (CHEWS), work at these facilities. There are often no doctors, but in some cases there are visiting doctors.

Most of the pharmacy shops are located in the urban area though a few are found in rural areas. PMDs are found in both urban and rural areas. There are regulatory bodies governing them namely the Pharmaceutical Association of Nigeria and Association of Patent Medicine Dealers, respectively. The State Ministry of Health has general oversight. Pharmacies and PMDs are licensed to sell over-the-counter drugs only. PMDs are retail outlets for drugs but also act as *de-facto* service providers.

To obtain a license, pharmacy shops are required to have at least one qualified pharmacist. In contrast, PMDs do not require any form of special training or qualification to obtain their license. Most of these private facilities obtain their drug supplies through both formal and informal channels including large retail and wholesale pharmacies in major cities, direct from pharmaceutical companies, and through visiting company representatives, a number of them from the open market.

All public facilities and medicine retailers in eligible clusters will be enumerated and facilities informed of the proposed study. Facilities will be selected at random and asked to provide written consent prior to cluster randomization. Where facility-level consent is not provided, replacement facilities will be randomly selected from the remaining list of eligible facilities. All providers responsible for diagnosis and treatment of suspected cases of malaria are eligible to participate in the provider survey and asked to provide written consent.

#### Patients/caregivers

All patients (or their caregiver) attending the health facilities and medicine retailers will be approached on exit for consent to participate in an exit survey and screened for their eligibility. Patients will be eligible if they are present at the facility and they (or their caregiver) report seeking treatment for fever or suspected malaria. Patients will be excluded if they are pregnant, less than 6 months old, or have signs and symptoms of severe malaria. The household survey will be administered with a household member (usually the mother as the main caregiver) and she (or he) will be asked about their knowledge and preferences relating to malaria diagnosis and treatment and also details of any treatment seeking in the past 2 weeks.

#### Schools

Primary and secondary schools in the study clusters will be eligible to take part in the study. There are a total of 67 secondary schools in both Enugu urban and Udi (that is 45 in Enugu Urban and 22 in Udi) and 247 primary schools in both Enugu Urban and Udi (that is 156 in Enugu Urban and 91 in Udi). On average, there are approximately three schools per community and virtually every community has a primary and a secondary school. In Enugu urban, some of these schools are in the same compound, bearing a similar name but differentiated by numbering (such as I, II, III, IV) and managed by different administrators commonly known as ‘head teacher’ in primary schools and ‘principal’ in secondary schools. For the purposes of this study schools within the same compound were considered as a single school in the selection process. This compound characteristic of the urban schools is not same in Udi as the schools are widely spread often located in different villages within the same community/intervention cluster.

Consenting schools will be invited to participate in a range of activities designed to raise awareness about diagnosing malaria using RDTs and that ACTs are the recommended antimalarial. With support from the research team, the head teachers and school principals with their deputies will oversee the intervention in their schools. In compound schools administrators for each school within the compound will oversee the intervention.

### Interventions

Intervention activities for this study will be: facilities invited to supply RDTs; demonstration on how to use RDTs; provider training and supervision; and school-based intervention.

#### Facilities invited to supply RDTs

RDTs for diagnosing malaria will be made available to all health facilities that participate in the study and attend the demonstration by the research team on how to use RDTs. The RDT that will be provided is SD Bioline Malaria Ag Pf, which was chosen in conjunction with the States Malaria Control Programme, and is reported to have a minimum detection rate for *P. falciparum* of 97.5% even at low levels of parasitaemia (200 parasites/μL) [30].

Estimates of RDTs required at each facility will be determined in discussion with the facility head and based on routine records for the number of febrile patients that a facility can expect during 1 month (taking into account seasonal variations) as well as data gathered during the formative research. The research team will procure adequate quantities of RDT kits to last throughout the evaluation phase. The project will also be responsible for distributing the RDTs to health facilities. Facilities will be able to request stocks from the research team when they run out or collect them from the research team’s office. Stock management records will be kept by the research team to monitor the distribution of RDTs. The RDT kits will be stamped for identification and facilities will be advised to store them in a cool, dry place. The research team has developed a commodity tracking system involving the use of stock issuing and stock receiving vouchers to keep track of the kits so they know when there is likely to be a stock-out.

Currently, the state government advises that RDTs should be available without charge in primary health centers for pregnant women and children under 5 years old. Hence, the project will not charge any fee for RDTs in the public sector. However, RDTs will be distributed to providers of private facilities at a subsidized cost of 50 Naira (US$0.3) per test^a^. Facilities are asked not to sell kits beyond 100 (US$0.6) to their clients so that the test can remain affordable. These facilities will reimburse the study team when they finish using the test kits. The study team will not supply ACTs to health facilities.

#### Demonstration on how to use RDTs

Providers will be invited to attend a demonstration on how to use RDTs. This is to ensure that providers are shown the steps involved in using the RDT and the important safety procedures, such as wearing gloves when conducting the test, how to prick a finger to get a sample of blood, and safe storage and disposal of the used materials. The demonstration will include a practical exercise in which the providers will each conduct a test, under observation. Participants will also receive a copy of the WHO job aid which shows the steps in using an RDT. The demonstration will be conducted by the Research Team in collaboration with the State Malaria Control Programme.

#### Provider training intervention (including support visits)

In addition to the supply of RDTs and the demonstration of how to use RDTs, facilities in clusters randomized to arms 2 and 3 will receive additional provider training and support visits. Provider training on malaria diagnosis and treatment will be conducted over 2 days and contains six training modules on: (1) Knowledge on malaria; (2) Introduction of the updated guidelines for malaria diagnosis and treatment; (3) Appropriate diagnosis; (4) Appropriate treatment when test is positive; (5) Appropriate treatment when test is negative; and (6) Effective communication. Together these training modules will improve providers’ knowledge and skills on why it is important to test for malaria, how to use a RDT, and the effective implementation of clinical guidelines. The first module describes the current burden of malaria in Nigeria, in addition to its causes, signs, and symptoms. The second module reviews the clinical guidelines and highlights the importance of malaria testing in febrile patients before treatment is prescribed. The module on appropriate diagnosis includes a practical session in which all providers will get hands-on experience of the steps involved in using an RDT. This is equivalent to the intervention ‘demonstration on how to use RDTs’ that is delivered to participants in Arm 1. Module 4 provides training on what treatment to give when a test is positive, the recommended types of antimalarial drugs, including the dosage regimens for different age groups and types of ACT. Module 5 provides advice on other causes of febrile illness which should be investigated if the malaria test is negative. The objective of the last module on communication is to improve health provider knowledge of the importance of patient communication and barriers to effective communication. Providers will learn how to discuss different treatment options with patients especially when the test result is negative.

Training will be conducted in different venues depending on the study site. In Enugu, the Laboratory of the Department of Pharmacology and Therapeutics, College of Medicine, University of Nigeria Enugu will be the venue. In Udi, the district hospital conference center and local government area headquarters hall will be used. The following types of providers will be invited to the training: in the public facilities, the officer in charge and one other health worker who is involved in prescribing treatment; and in the private, the head of the facility or whoever s/he appoints. It is anticipated that two providers from each public facility and one from each private facility will attend. The training will be conducted by eight people from the research team and four people from the state malaria control programme. The trainers will receive extensive briefing by the research team and be given a trainers’ manual in addition to the participants’ manual which provides details of the material for each module and how it should be delivered.

Each training workshop will aim to train 20 to 25 providers. The training primarily takes a seminar style in which the trainer delivers the training material, though there will be discussions, practical sessions, and question and answer sessions using short case scenarios. A participants training manual will be given to providers that attend the training course and this includes all essential reference material such as the malaria treatment guidelines. Participants will also be provided with job aides on how to perform RDTs, a treatment algorithm which can be displayed in their facilities and a poster describing all categories of nationally recommended drugs for the treatment of malaria, their generic names, and dosage regimens. While not enforced, all participants of the provider training will be strongly encouraged to train others who are involved in malaria treatment at their facilities.

Members of the research team will provide support visits to each facility every month during the implementation phase (3 months) and the subsequent evaluation phase (approximately 2 months) to monitor and assess what they are doing and to reinforce the skills acquired by providers during the earlier training workshops. During the support visits, where possible, providers will be observed delivering treatment to patients who have sought treatment for fever, and questions on the different aspects of the training will also be asked. Based on the responses, guidance will be provided on areas where the provider is experiencing difficulty. Providers will also be asked about any challenges implementing what they were taught during the training.

#### School-based intervention

This intervention will be implemented in selected primary and secondary schools in communities randomized to arm 3. Peer health education has been shown to influence the knowledge, attitudes, and practice of school children and their families as well as the wider community [31]. In Enugu State, school-based health education helped to improve community awareness and participation in onchocerciasis control activities [32-34]. In Ghana [35], Lao PDR [36], and Thailand [37], school-based malaria interventions have also been shown to improve overall control of malaria within the communities where the schools were located.

One of the documented advantages of school-based interventions is their ability to reach a relatively large proportion of any given community [38]. The reach of a school-based intervention in Nigeria is expected to be comparable. About 75% of households have school-aged children (either their direct children or wards) and about 44% of school-aged children (6–17 years) are in schools (50% in primary school, 42% in junior secondary school, and 36% in senior secondary school) [32].

The research team will train two teachers per school (one health teacher and one social teacher) who will in turn train six school children as peer health educators (PHEs) with the support from the research team, giving 130 teachers and 390 peer-health educators in total. The PHEs will be responsible for implementing a range of activities designed to raise awareness about diagnosing malaria using RDTs and that ACTs are the recommended antimalarial. Activities including dramas, songs, card games, and health talks, will be undertaken during morning assembly, Parent Teachers Association (PTA) meetings, and at some school events such as prize-giving days. In addition, teachers and PHEs are supported to hold their own school malaria events involving parents, guardians, and other community members that will be invited to participate in card games, dramas, songs, and health talks. Handbills, posters, T-shirts, and baseball caps promoting the school-based intervention will be distributed at all events. A short description of each type of activity is given below.

A short drama will emphasize the rational use of antimalarial drugs, including the use of ACTs and the need to test before treatment. The school children will perform the drama in school. Each drama session will last for no more than 15 minutes. Transportation and costumes will be procured by the research team and T-shirts will be given to the drama team with the inscription ‘REACT AGAINST MALARIA’. A drama sketch has been developed by a local theater artist for training purposes.

The research team provides teachers with malaria songs that they in turn will communicate to PHEs. The songs will emphasize the need to go for a test when one has a fever or headache and to take an ACT when the test is positive. Three different songs have been composed by local artists. Each of the songs will last for 5 minutes and contain up to four verses.

A card game will be introduced to school children and community members, which teaches and reinforces components of appropriate treatment of malaria. Between four and six participants take turns in collecting cards and achieve a point when they present three cards that show a patient has received treatment in line with guidelines. This can be achieved by presenting a ‘patient with fever’ card accompanied by a ‘RDT positive’ card and an ‘ACT’ card, or alternatively by presenting a ‘patient with fever’ card accompanied with an ‘RDT negative’ card and a ‘further investigation’ card. The game ends when a participant has treated five patients in line with the guidelines and scored 5 points.

Health talks will be given by the PHEs to the schoolchildren in selected primary and secondary schools in the intervention clusters. The health talk will include issues about appropriate treatment of malaria including the need to have a malaria test before taking treatment, asking for ACTs when the malaria test is positive, not asking for an antimalarial when the malaria test is negative, not to take monotherapies, and and the importance of sharing the knowledge they have gained with other members of their households.

PHEs will place posters in classrooms, head teachers’ offices, staff common rooms, assembly grounds, and other strategic places. Handbills will be shared with community members during malaria events in schools and also be given to providers at the facilities so they can distribute to patients who visit the facilities. The posters will also be displayed at health facilities and at prominent places in the intervention clusters such as market places, village squares, and village halls. The key messages contained in the posters and handbills include steps towards appropriate treatment of malaria (that is the need to have a diagnostic test before taking malaria treatment; people should ask for or receive ACTs when a test is positive; people should not receive an antimalarial when a test is negative; and people should not receive monotherapy).

The research team will conduct support visits to each school every month during the implementation phase and the subsequent evaluation phase to guide and encourage teachers and PHEs involved in the school-based malaria education. During these visits they will check to see if teachers have created PHEs and if possible attend a meeting of the PHEs. Where PHEs have not been set up, the research team will encourage and support their establishment. Also during support visits, the team will review preparations for the school malaria event and attempt to observe the drama group rehearsals and health talk presentations, and check if posters have been displayed.

### Objectives

The primary objectives are as follows:

1. To evaluate the effectiveness and cost-effectiveness of the provider intervention compared to expected standard practice. Where:

(i) expected standard practice is defined as facilities having access to RDTs and have been shown how to use them

(ii) provider intervention is defined as provider training and supervision on malaria diagnosis and treatment in a setting in which facilities can offer rapid diagnostic testing (including a demonstration on how to use RDTs);

2. To evaluate the effectiveness and cost-effectiveness of the combined provider intervention and the school-based intervention to expected standard practice; and

3. To evaluate the effectiveness and cost-effectiveness of the combined provider intervention and the school-based intervention compared to the provider intervention alone.

Secondary objectives include:

1. To describe the process of implementing the interventions including participant assessment of the training received by providers and teachers;

2. To evaluate the impact of interventions on provider knowledge and ability to test and appropriately treat patients with suspected malaria;

3. To evaluate the impact of interventions on community knowledge of and preference for malaria diagnosis and treatment;

4. To evaluate patient satisfaction with the quality of care received at the health facility;

5. To calculate the economic and financial costs of the interventions;

6. To assess whether the effectiveness and cost-effectiveness of the interventions vary according to urban/rural residence or the socioeconomic status of the patient.

### Hypotheses

1. The provider intervention will be more effective and cost-effective in improving the treatment and diagnosis of malaria compared to what is expected to be standard practice.

The components of the provider intervention will lead to the delivery of more appropriate treatment. Specifically, the provider intervention will improve the competency of providers to deliver treatment to febrile patients based on the result of a malaria test.

2 The combined provider and school-based intervention will be more effective but also more costly compared to expected standard practice and the provider intervention alone.

The school-based intervention will have a direct effect by improving the knowledge of community members in terms of why they should be tested, the availability of testing, and that ACTs are recommended for confirmed cases of malaria. We expect that these changes in knowledge will also affect what patients ask for when they attend health facilities, and are therefore more likely to ask for a test and/or ask for an ACT.

The school-based component of the community intervention will also have an indirect positive effect on provider practices. Specifically, if providers are aware of the school-based intervention, they will know that patients have been made aware that malaria testing is available and recommended, and that malaria should be treated with an ACT. It is hypothesized that the providers will then feel more comfortable suggesting that patients are tested and confident in recommending that confirmed cases should be given an ACT.

While the combined interventions are predicted to have a positive synergistic effect, the combined costs of a school-based intervention in both primary and secondary schools and of provider training workshops that involve supervisory visits are likely to be greater than those associated with expected standard practice or the provider intervention alone.

**Figure 1:**
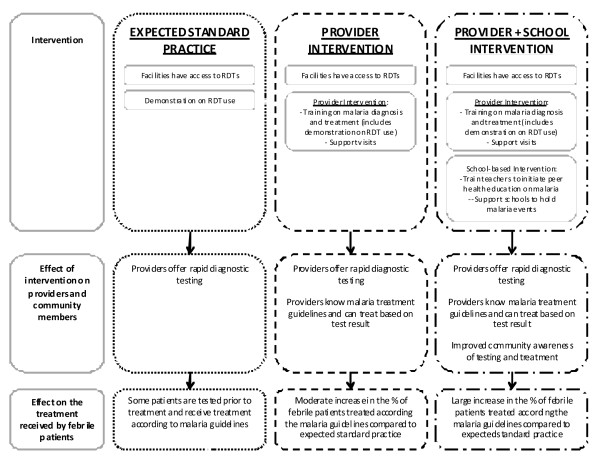
Effect of interventions on the treatment received by patients.

The relationship between the study hypotheses and outcomes are summarized in Figure 1.

### Outcomes

#### Primary outcome

The primary outcome is the proportion of patients attending facilities that report a fever or suspected malaria and receive treatment according to malaria guidelines. The corresponding measure of cost-effectiveness is the cost per febrile patient that receives treatment according to the malaria guidelines.

Treatment according to the malaria guidelines is a composite endpoint requiring that: febrile patients should be tested for malaria, using either microscopy or an RDT; the patient should receive an ACT if s/he has a positive malaria test result; and the patient should not receive an antimalarial if s/he has a negative malaria test result.

**Figure 2:**
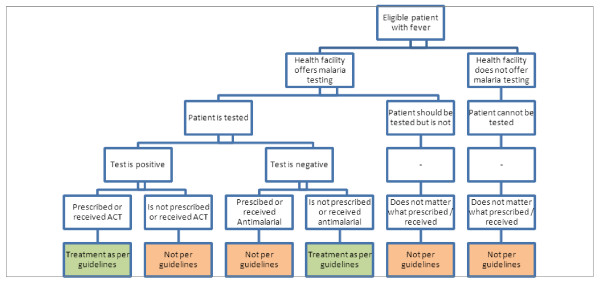
Primary outcome measure.

The outcome measure is summarized in Figure 2.

#### Secondary outcomes

Secondary outcomes include the following:

1. Patients: proportion of febrile patients that are tested for malaria; proportion of febrile patients receiving an antimalarial that receive an ACT; proportion of febrile patients receiving an ACT that receive the correct dose for their age; proportion of febrile patients receiving an ACT that accurately report how to take the medicine; proportion of febrile patients that report they are satisfied with the care received; proportion of patients attending a health facility that report a school malaria event took place in their community.

2. Providers: proportion of providers that report they were satisfied with the training received; proportion of providers that report febrile patients should be tested for malaria; proportion of providers that know how to identify positive, negative, and invalid malaria RDT results; proportion of providers that know ACT should be given if the malaria test is positive and that an antimalarial should not be given if the malaria test is negative; proportion of providers that know the correct dose of the first line ACT in an adult and in a child aged 2 years.

3. Community members: proportion of individuals that were aware of a school malaria event; proportion of individuals that report they had attended school malaria event; proportion of individuals that report febrile patients should be tested for malaria; proportion of individuals that know ACT is the recommended treatment for malaria; proportion of individuals that know ACT should be given if the malaria test is positive and that an antimalarial should not be given if the malaria test is negative.

4. Costs: total cost of the provider and school-based interventions; mean cost per provider trained under the provider intervention; mean cost per school participating in the school-based intervention.

Secondary outcomes related to patients will also be reported in terms of their urban/rural residence and socioeconomic status.

### Evaluation design

The evaluation of the interventions will use data collected in a patient exit survey, a register of malaria tests conducted by the provider during patient consultations, a provider survey, documentation of the intervention process, a household survey, and costing of the intervention activities. The patient exit survey will be administered before the provider survey to ensure that the treatment received by patients is not influenced by the content of the provider questionnaire. Each of these research instruments is described below.

#### Patient exit survey

The primary outcome will be measured through an interviewer-administered patient exit survey. Data collection will commence 3 months after the intervention has been implemented. The three-month lag before data collection is to ensure that the effect measure reflects treatment practices in the medium term. In the short term it is recognized that it is possible that the effect is overstated because providers change practices initially but revert to past behaviors over time, or that the effect is understated because it takes time for the training to have an effect as some providers are hesitant and want to learn from the experience of the early-adopters.

The research team will recruit field workers and provide training over 1 week on all aspects of data collection related to the patient exit survey. The training will include a practical assessment of their ability to provide information to respondents about the survey, obtain consent and administer the questionnaire. The research team will supervise the field workers and will accompany the field worker at the start of data collection to obtain consent from the head of the facility and ensure the fieldworker adheres to the standard operating procedures. Supervisory visits to monitor the performance of the field workers will take place at least once each week during the data collection period.

The patient exit questionnaire is designed to collect information about the patient’s experience of seeking treatment and has been piloted at selected facilities in the study site. The questionnaire contains the following 10 modules:

A. Background Information, Consent and Screening Questions

B. Details of the Respondent and/or Patient

C. Reasons for attendance

D. Consultation and diagnosis

E. Treatment prescribed and received

F. Patient satisfaction and knowledge of malaria

G. Costs of seeking treatment

H. Household characteristics

I. Malaria test completed by the research team (in sub-sample of patients)

J. Malaria test completed by providers (from register of malaria tests at facility)

#### Register of malaria tests conducted

The patient exit questionnaire will be supplemented by a register of malaria tests at each participating health facility because patients may not always know if they were tested for malaria and the result of the malaria test. With consent from the head of the facility, providers responsible for conducting malaria tests will be asked to keep a register of all malaria tests undertaken. The following data will be collected: details of the patient, availability of microscopy and RDT, method of test conducted, test result and the provider that conducted the test. At each facility the field workers will collect the register of malaria tests at least once each week and will use the patient’s name, gender, age, and date of visit to identify the patients that completed the survey and record the details in Section J of the questionnaire.

#### Provider survey

The research team will administer a survey to all providers responsible for the diagnosis and treatment of suspected cases of malaria. Providers are eligible to participate if their responsibilities include any of the following activities: taking patient signs and symptoms, undertaking diagnostic tests, prescribing or dispensing medication.

The provider survey has been designed to collect data on the providers’ characteristics, knowledge and preferences for diagnosing and treating malaria and details of the resources available at the health facility. The survey will be piloted with providers at facilities that are not participating in the study. The questionnaire contains the following modules (of which A and B are completed by all providers and C to G are completed once for each facility):

A. Background information, consent and screening questions

B. Provider characteristics and treatment practices

C. Details of the health facility

D. Management and procurement of drugs

E. Availability of RDTs

F. Availability of antimalarial drugs

G. List of all providers that are involved in diagnosis or treatment

#### Documentation of the implementation of the intervention

The process of distributing the RDTs to health facilities will be monitored and any problems with the procedures for replenishing RDT stocks will be documented. Similarly, the occurrence of stock-outs of ACTs in all the facilities will be monitored.

Details of all participants attending the provider training on malaria diagnosis and treatment will be recorded. Participants will undertake a pre- and post-training test to determine the impact of the course on their knowledge of malaria diagnosis and treatment. In addition, all participants will be invited to complete the training evaluation, which assesses the content and delivery of the training course. The trainers will also complete a form to record any challenges faced in running the training workshop.

For the school-based intervention, details of all participants attending the training course will be recorded, and copies will be taken of the action plans developed during the training course. All course participants will be asked to complete an evaluation form and assess the content and delivery of the training course. All participants will also be asked to complete a pre- and post-training test which will indicate the effect of the course on participant’s knowledge of malaria and peer health education.

The extent to which the school-based intervention is implemented by teachers in schools will be recorded by a representative from the research team who visits the school once the training is complete to provide support for the preparation of the school-based malaria intervention. During this visit the representative will note progress (with reference to the action plan developed by the teachers). The representative will also attend the school-based malaria event and record the activities held and attendance rate.

Implementation of the interventions is expected to vary by provider and/or by school thereby reflecting what would happen if the government were to roll out the interventions in a ‘real life’ setting. Data on the implementation process will reveal factors affecting compliance.

#### Household survey

A household survey will be undertaken to collect data on the community knowledge of malaria diagnosis and treatment, and on experience of treatment seeking for febrile illness that had been experienced in the previous 2 weeks. This will provide insight into the reach of the school-based activities and the effect of the intervention on the knowledge and preferences of community members. Data collection will commence 3 months after the intervention has been implemented. As mentioned earlier, the three-month lag in the data collection is to ensure that the effect measured reflects treatment practices in the medium-term.

The household questionnaire will be completed by one individual per household, usually the primary caregiver. Modules B and C will be asked of several individuals in each household to consider whether there are differences in the knowledge of malaria diagnosis and treatment by respondent characteristics.

The household survey contains the following modules:

A. Background information

B. Household members

C. Household knowledge of malaria and malaria treatment (including attendance at school malaria event)

D. Treatment seeking of each household member with a fever in the past 2 weeks (if applicable)

E. Household characteristics

#### Costs

The direct and indirect costs of each phase of the interventions (that is development, implementation, upkeep) will be assessed from both a provider and societal perspective using standard economic evaluation methodologies [39]. Cost data will primarily be estimated from health facility records, project financial accounts, and from the provider and patient exit surveys. An estimate of healthcare savings will also be included and subtracted from costs using the Shillcutt model [22].

### Quality assurance

#### Data collection and management

There is a quality assurance officer responsible for ensuring all implementation and evaluation activities adhere to standard operating procedures. Quality assurance will include monitoring the process of obtaining consent, data collection, transfer of completed survey instruments, data management, and the secure storage of study materials. In addition, field supervisors will monitor the survey administration undertaken by field workers and make frequent visits (at least once a week) to assess the quality of data collection and review completed questionnaires.

Only authorized staff with appropriate training will have access to the databases to perform data entry. All databases will be password protected. Each data form will be entered by two data entry clerks in a database of the same structure using two different computers. Entries will be compared for discrepancies using the Epi info 2000 data compare utility. Any discrepancies will be corrected by crosschecking against the corresponding original questionnaire. Checks (validation rules) will be implemented in different fields of the database. Data will also be queried electronically to ensure the correct data are entered under the correct variables for each section of the form/questionnaire. A log of all data changes will be kept. Questionnaires will be kept in a locked filing cabinet.

#### Independent verification of malaria tests conducted and test results

Reliance on providers’ register of malaria tests conducted and their interpretation of the test result may be a risk for data quality. For example, we are dependent on the providers’ skills in conducting and interpreting the test results and the accuracy of their record-keeping. We will examine the accuracy of the register of malaria tests by comparing the patient reported data on whether they had a test with the register. We will also independently conduct RDT tests in a subsample of 5% of patients on exit that reported they were tested for malaria to determine the degree of consistency between the test result recorded by the provider and the test result conducted by the fieldworker. Quality assurance of the RDTs is beyond the scope of the study.

### Sample size

#### Patient exit survey

Sample size calculations are based on the primary outcome; the proportion of febrile patients receiving treatment as recommended in malaria treatment guidelines. Based on provider adherence to test results in our formative research and assumptions about the incentives of providers in private facilities we expect that this will be 10% in the control arm (basic provider training) with a coefficient of variation within stratum of 0.35. Using methods for stratified cluster randomized trials [40] 14 clusters per arm with a harmonic mean of 50 febrile patients per cluster will be needed to detect a 15% incremental increase between the study arms, from 10% to 25% for the provider intervention (arm 2), and from 25% to 40% with the addition of the school intervention (arm 3) with 80% power at a 5% significance level.

#### Provider survey

The sample size calculations for the provider survey gives the anticipated level of precision for calculating the proportion of providers that know the treatment guidelines (that is report that parasitological testing is recommended and that ACTs are for confirmed cases of malaria). Based on logistics and costs a total of 138 facilities (an average of three to four facilities per cluster) will be included in the study. From the baseline survey we know that there are three to four workers per facility hence we can expect on average 138 workers to be surveyed per arm. Assuming that the estimate of the primary outcome is 50% in the control arm and 75% in each of the intervention arms, and an intra-correlation coefficient (ICC) of 0.25, this allows us to estimate the true primary outcome with ± 14.5% precision in the control arm and ± 12.5% precision in each of the intervention arms.

#### Household survey

\Sample size calculation for the household survey is based on the number of clusters identified for the patient exit survey (that is 14 clusters per arm) and gives the anticipated level of precision for calculating the primary outcome; the proportion of individuals surveyed that know that parasitological testing is recommended and know that ACTs are the recommended treatment for confirmed cases. Assuming that the estimate of the primary outcome is 10% among those attending facilities in Arm 1 (expected standard practice), 20% for those living in clusters receiving the provider intervention (Arm 2), and 50% in the combined provider and school intervention clusters (Arm 3), that one individual per household will be sampled and the intra class-correlation (ρ) will be 0.2, we will survey 25 per cluster (350 per arm). This allows us to estimate the true primary outcome with ±7.9% precision in Arm 1, ±10.5% precision in Arm 2, and ±13.1% precision in Arm 3.

### Randomization

With cluster randomized trials there is an increased chance that the study arms are unbalanced with respect to known and unknown potential confounders, and therefore undermines the credibility of the trial results. Stratified randomization will reduce some of the imbalance in factors known to be correlated with the study outcome and the study site. However, this does not enable us to balance on type of facility and the number of facilities which were also expected to be important correlates of the primary outcome. Hence, a system of constrained randomization [41,42] will also be used to allocate communities to the study arms within each stratum to ensure balance in these factors.

The validity of the restricted randomization will be assessed by producing a matrix where the rows and columns represent the clusters and the elements of the matrix are the proportion of times each pair of clusters is allocated to the same study arm, that is the probability that the i^th^ cluster is being allocated to the same intervention group as the j^th^ cluster. The matrix will then be examined for under- and over-represented pairs that would highlight any potential causes for concern in the randomization.

**Figure 3:**
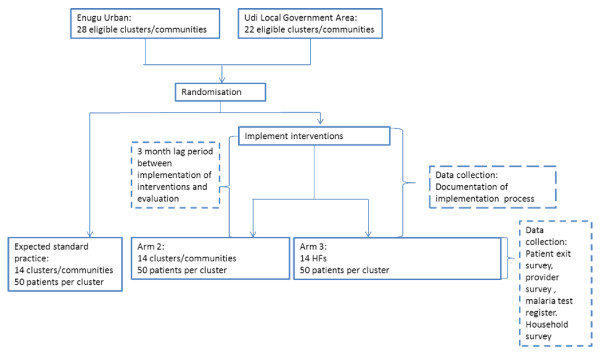
Eligibility, selection, enrolment, and data collection.

Randomization of the clusters will be performed by the study statistician after informed consent has been sought from the heads of the facilities, community heads, and schools to avoid selection bias. Patients (or caregivers) and fieldworkers administering the patient exit survey will be blinded to group assignment. The research team involved in implementing the interventions and supervising data collection will need to be aware of which clusters and in turn which facilities and schools receive the different interventions (Figure 3).

### Data analysis

The primary outcome will be compared between each of the study arms using methods appropriate for cluster randomized trials. Due to the small number of clusters per arm analysis based on cluster-level summaries will be applied. Point estimates of the primary outcome will be calculated using the weighted average of the cluster proportions, with the weights provided by the sample size for each cluster. If the distribution of the summary measures in each study arm is skewed, a logarithmic transformation to the proportions will be considered. An overall estimate of the risk difference will be obtained by taking a weighted average of the stratum-specific estimates with the weights proportional to the number of clusters in each stratum since an equal number of clusters have been allocated to the study arms within each stratum. 95% confidence intervals (CI) will be adjusted for observed between-cluster variance and formal hypothesis testing will be conducted using stratified t-tests. Adjustment for covariates, including patient and provider characteristics and knowledge, contextual factors, and process factors, will be carried out using a two-stage process. In the first stage a logistic regression model including stratum as a fixed effect and the covariates of interest, but excluding the intervention effect, will be fitted to calculate cluster-specific expected values. The difference of observed to expected values will give the difference-residual for each cluster. In the second stage, the above methods are carried out with the cluster-level proportions replaced with the covariate-adjusted residuals.

Secondary outcomes will be analyzed using the methods described above. To examine whether secondary outcomes associated with the patient vary according to urban/rural residence and socioeconomic status methods appropriate for examining an interaction between the intervention and the individual-level variable will be applied [43].

Data will be double entered using Microsoft Access 2007 (Microsoft Inc., Redmond, WA, USA) and analyzed using STATA version 11.0 (STATA Corporation, College Station, TX, USA). A full analysis plan will be reviewed and agreed before the data are analyzed.

#### Cost-effectiveness

Cost-effectiveness ratios will be based on the primary outcome (that is the cost per case of suspected malaria that received treatment as recommended in the malaria guidelines) as well as a range of secondary outcomes including changes in provider knowledge. Cost-effectiveness will be calculated for each comparison and will be expressed as incremental cost-effectiveness ratios (ICERs). One-way and multiway sensitivity analysis will be undertaken to examine the effects of varying uncertain variables on study findings. Costs and effects will be presented in both discounted and undiscounted form.

### Dissemination

Results from the study will be reported at local, national, and international levels. At the local and national level, the Research on the Economics of ACTs (REACT) Project (http://www.actconsortium.org/pages/project-5.html) will continue working with the Ministry of Health after the trial is completed to adapt the most cost-effective interventions for national use. At the international level, we also see an opportunity to support the implementation of the 2010 WHO malaria treatment guidelines which acknowledge the need for provider training alongside the large-scale deployment of RDTs and ACTs.

## Trial status

The trial is ongoing. Patients are still being recruited.

## Endnote

^a^Prices are based on a willingness to pay study undertaken in Enugu State by Uzochukwu *et al*. [38].

## Abbreviations

ACTs: Artemisinin-based combination therapies; RDTs: Rapid diagnostic tests; REACT Project: Research on the economics of artemisinin-based combination therapies.

## Competing interests

The authors declare that they have no competing interests.

## Authors’ contributions

VW, OO, and BSC secured the funding and are responsible for the overall study design and project management. OO, EO, NE, EJC, and UE were responsible for coordination and supervision of fieldwork. LM participated in the study design and overall study coordination. OO, EO, NE, and LM designed the provider interventions. NE, EJC, OO, BSC, and LM designed the community intervention. BC led the statistical design. OO and EO coordinated data entry and management. All authors contributed to the original protocol while VW drafted the manuscript. All authors read and approved the final manuscript.
